# Hunger affects cognitive performance of dairy calves

**DOI:** 10.1098/rsbl.2022.0475

**Published:** 2023-01-18

**Authors:** Benjamin Lecorps, Raphaela E. Woodroffe, Marina A.G. von Keyserlingk, Daniel M. Weary

**Affiliations:** ^1^ Bristol Veterinary School, University of Bristol, Langford House, Langford, Bristol BS40 5DU, UK; ^2^ Animal Welfare Program, Faculty of Land and Food Systems, University of British Columbia, 2357 Main Mall, Vancouver, BC V6T 1Z6, Canada

**Keywords:** emotion, milk restriction, weaning, cow, well-being

## Abstract

Hunger remains a significant animal welfare concern as restricted feeding practices are common on farms. Studies to date have focused on negative effects on health and productivity but little research has addressed the *feeling of hunger*, mostly due to methodological difficulties in assessing animals' subjective experiences. Here, we explored the use of a cognitive approach to disentangle motivational hunger (a normal state that is of limited welfare concern) from distressful hunger (a state associated with intense negative emotions). Cognitive performance in a foraging task is expected to follow an inverted U relationship with hunger levels, providing an opportunity to make inferences about different hunger states. We assessed the effect of milk restriction on calf cognition in two experiments using a modified hole-board test. Experiment 1 showed that reducing milk allowance from 12 to 6 l d^−1^ impaired all measures of cognitive performance. Experiment 2 showed that the same type of feed restriction also disrupted calves’ capacity to re-learn. We conclude that hunger associated with reduced milk allowance can disrupt cognitive performance of dairy calves, a result consistent with the experience of distressful hunger.

## 1. Introduction

Freedom from hunger is one of the pillars of animal welfare, but many farm animals routinely experience feed restriction [1]. Hunger is associated with affective states [2] of negative valence and high arousal in humans [3], but the *feeling of hunger* remains largely unexplored in non-human animals [4]. Given that subjective experiences are increasingly seen as core to animal well-being [5], a better understanding of what animals experience when subjected to feed restrictions is needed.

Dairy calves are subjected to reductions in milk allowance when weaned from milk to solid feed, a process that occurs at an earlier age and more abruptly on most dairy farms compared to the natural process [6]. Most research to date has shown that low milk allowances and abrupt weaning negatively impact health and productivity [7]. Behavioural changes, including increased vocalizations [8] and visits to the milk feeder [9] have been used to draw inferences regarding feelings of hunger. Such changes reflect increased motivation to access food but do not allow strong inferences about the affective experience *per se* [10]. Food motivation may be driven by a negative emotion animals seek to relieve (distressful hunger) or because they seek the positive experience (motivational hunger) associated with food consumption [11]. These two distinct emotional experiences have different implications for animal welfare.

Hunger is associated with higher performance in foraging tasks. For instance, fasted guppies are quicker at learning the path to a new feeder [12]. However, according to the regulatory depletion hypothesis, more hunger can have detrimental effects, most notably because it decreases self-control leading to poorer decision-making [13]. Thus an inverted, U-shaped relationship between hunger and cognitive performance is expected, with high levels of hunger associated with poorer cognitive performance.

We tested this hypothesis using a hole-board test adapted for dairy calves. In this spatial foraging task, animals must remember the location of four rewards (bottles ‘baited’ with milk) among 15 possible locations (empty bottles) [14]. This test assesses several aspects of cognition including working memory (how good animals are at avoiding revisits to locations they already visited within trials) and reference memory (how good animals are at remembering the baited locations between trials) when bait location is kept constant, as well as assessing behavioural flexibility (how well animals re-learn) when baited locations are changed [15].

We explored whether a sudden milk restriction (reduced from 12 to 6 l d^−1^) negatively affects cognitive performance when bait location were not changed (Experiment 1), and would disrupt the capacity to re-learn after changing locations of the baited bottles (Experiment 2). A 50% reduction in milk allowance was chosen because it is used in stepdown weaning protocols [16] and has been found to induce behavioural signs of hunger [15]. The two experiments were done with separate cohorts of calves. In both cases, calves were initially tested in the hole-board test for 12 days (in the morning) when fed their standard milk allowance (12 l d^−1^). On day 12 (after participating in the hole-board test) and following days, milk allowance was reduced to 6 l d^−1^. This was applied to all calves in Experiment 1 (within-individual design) and to half the calves in Experiment 2 (to assess the effect of bait location change in feed-restricted and non-restricted calves). The effect of this change in milk allowance was assessed in calf performance on the hole-board test from days 13 to 18. We expected calves who experienced a sudden reduction in milk allowance to display poorer cognitive abilities (Experiment 1) and to show learning deficits (Experiment 2).

## 2. Material and methods

Information about animals used and housing can be found in the electronic supplementary material [17].

### (a) Hole-board testing

A full account of pre-testing procedures can be found in the electronic supplementary material. Bottles were placed on three sides of an arena, with five bottles per side; of the 15 bottle locations just four were baited with milk (0.5 l each; providing a total of 2 l). Calves were tested individually for 18 days, always between 08.00 and 10.00 h. After testing calves had access to 10 l d^−1^ via automated feeders. The automatic feeders were programmed to allow milk access from 10.00 to 22.00 h; no milk was available overnight before the next morning's test session. Although calves experienced at least a 10 h of milk restriction before testing, some calves may have been fasted for longer depending on when they last drank on the day before testing.

During the hole-board test, a ‘visit’ was scored when the calf touched the nipple of any bottle with their muzzle. Visits were scored as ‘rewarded’ if the calf was observed to drink milk. Working memory (the number of rewarded visits divided by the total number of visits to the baited set), general working memory (the number of different bottles visited divided by the total number of bottles visited) and reference memory (the total number of baited bottles visited divided by the total number of bottles visited) were assessed for every trial [15]. Revisits to baited bottles that were not previously emptied were counted as rewarded. We also recorded the number of vocalizations.

In Experiment 1, baited locations were kept constant for 18 days. In Experiment 2, locations were changed on day 13. In both experiments feed restriction was applied on day 12 just after hole-board testing so that the effect of milk restriction was assessed on the following day (day 13). For this, milk allowance in the home-pen was reduced to 4 l on day 12 (at an average ± s.d. calf age of 38 ± 4.0 days in Experiment 1 and 32 ± 3.1 days in Experiment 2). Only half the animals were feed-restricted in Experiment 2, with treatment allocation alternated based upon calf age such that every other calf was enrolled in one of the two treatments.

### (b) Statistical analysis

Statistical analyses were run using SAS (v.9.4; SAS Institute Inc., Cary, NC) with mixed models and calf identified as a random factor. In all cases, normality of residuals was confirmed graphically. The number of vocalizations was Poisson distributed so we used a general linear model specifying this distribution. Trials durations are shown in electronic supplementary material, figure S1.

#### (i) Experiment 1

Four models (one per outcome: working memory, general working memory, reference memory and number of vocalizations) were used to explore the acute effect of feed restriction, comparing measures from day 12 with those on day 13 (i.e. the test sessions just before and just after the first day of reduced intake).

Although the milk allowance was set to 12 l d^−1^ before day 12, calves varied in their actual intake; calves drinking the most milk before this day thus experienced the largest reduction in intake. To test this effect, we used Spearman's correlation to compare the size of the decline in intake between day 11 and day 12 (i.e. first day of milk decline) and changes in responses to the hole-board between days 12 and 13.

We also assessed whether cognitive performance recovered by modelling changes in performance between days 13 and 18 (with day as a continuous factor).

#### (ii) Experiment 2

To assess the effect of the change in location of baited bottles, and how responses to this change were affected by the reduction in milk allowance, we ran models comparing measures on days 12 and 13, testing the effects of day, treatment and their interaction. To explore the effects of treatment on re-learning, we ran models exploring changes over time in calf performance assessing the effects of day (days 13–18, treated as continuous), treatment and their interaction.

## 3. Results

### (a) Experiment 1

**Figure 1.**
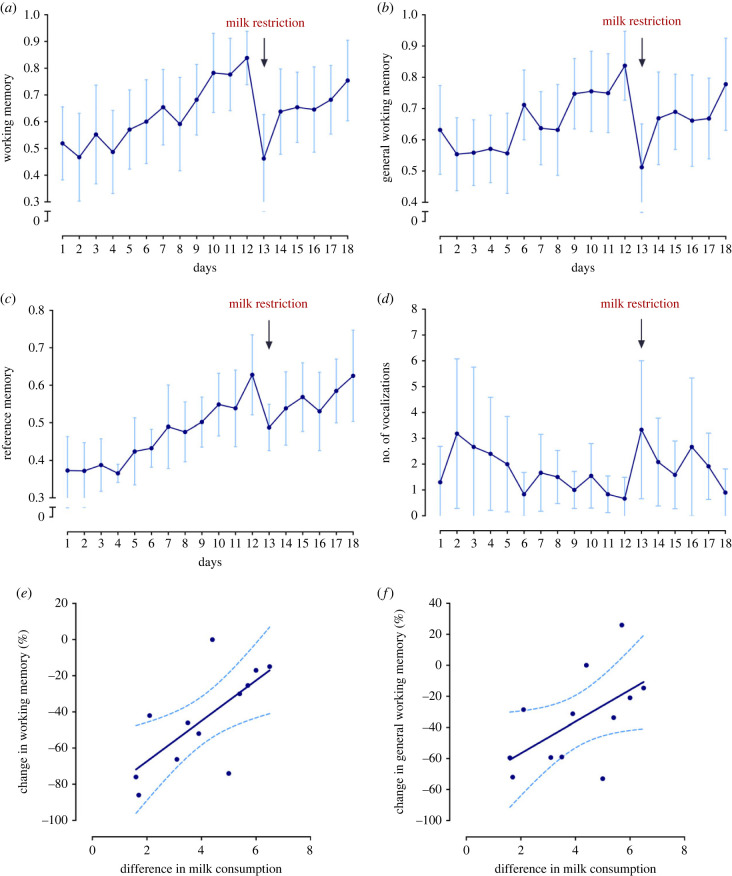
Changes in cognition and vocalization over 18 days of testing in a modified hole-board test (*n* = 12). Calves were trained to find four baited bottles pseudo-randomly located among 15 bottles for the first 12 days of testing. After the training session on day 12, milk allowance was reduced from 12 to 6 l d^−1^, such that test session from day 13 onwards were under conditions of milk restriction. Working memory (*a*), general working memory (*b*), reference memory (*c*) and number of vocalizations expressed (*d*) were assessed daily. For (*a*–*d*) dots show the mean and bars show ± 95% Cl. The change in working memory (*e*) and general working memory (*f*) after milk restriction was related to the magnitude of the decline in milk consumption. Negative change in performance (expressed in %) means calves revisited more baited bottles (working memory) and more bottles overall (general working memory) on day 13 compared to day 12. Dashed lines represent 95% CI.

The reduction in milk allowance impaired cognitive performance on day 13 (figure 1*a–d*). Consistent with reduced focus on the task, calves showed lower working memory (95% Cl = −0.21 to −0.55, *F*_1,11_ = 23.55, *p* < 0.001) and general working memory scores (95% Cl = −0.15 to −0.51, *F*_1,11_ = 15.98, *p* < 0.01). Calves also struggled to remember where baited bottles were located, as illustrated by lower reference memory scores (95% Cl = −0.024 to −0.26, *F*_1,11_ = 7.10, *p* = 0.022). The reduction in milk allowance was also associated with an increase in the number of vocalizations (95% Cl = 0.35–2.80, *F*_1,11_ = 8.10, *p* = 0.016). Illustrative videos (before and after milk restriction) can be accessed in the electronic supplementary material.

The magnitude of change in milk intake between days 12 and 13 varied among calves (from 1.6 to 6.5 l), driven by differences in baseline intake; milk intake averaged (±s.d.) 9.81 l (±1.34) on day 11 versus 5.73 l (±0.56) on day 12 (the day of feed restriction). Larger declines in intake were associated with a smaller reduction in working (*r*_S_ = 0.74, *p* < 0.01) and general working memory scores (*r*_S_ = 0.59, *p* = 0.046; figure 1*e*,*f*), showing that calves who drank more on the day before feed restrictions were less affected by the decline. This effect was not driven by differences in baseline performance (i.e. calves who drank more did not have lower baseline cognitive performance; all *p*s > 0.1).

Performance measures returned to baseline levels over days 13–18 (WM: *F*_1,57_ = 6.58, *p* = 0.013; GWM: *F*_1,57_ = 5.57, *p* = 0.022; RM: *F*_1,57_ = 4.70, *p* = 0.03) but number of vocalizations did not (*p* > 0.05). Together, these results are consistent with the experience of hunger (or its effects on cognitive performance) waning over time.

### (b) Experiment 2

**Figure 2.**
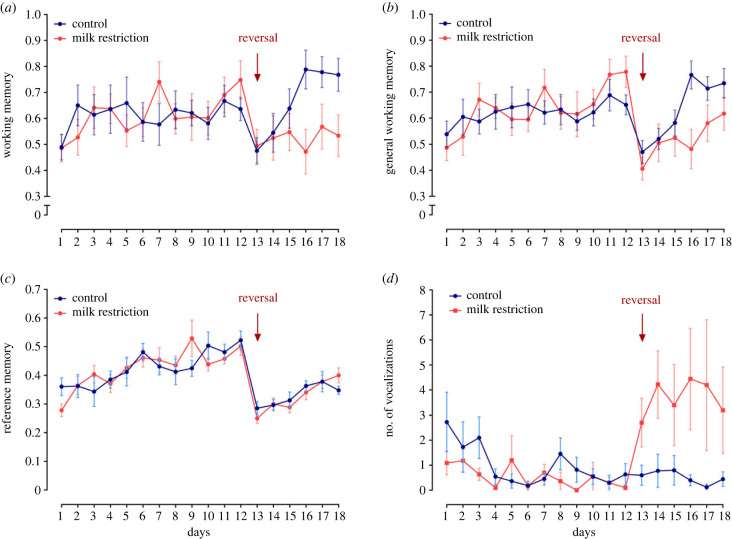
Changes in cognition and vocalizations over 18 days of testing in a modified hole-board test (*n* = 22). Working memory (*a*), general working memory (*b*), reference memory (*c*) and number of vocalizations expressed (*d*) were assessed daily. Calves were trained to find four baited bottles pseudo-randomly located among 15 bottles for the first 12 days of testing. On day 13, all calves experienced a change in the location of baited bottles (reversal). For half the calves (control) milk allowance remained constant at 12 l d^−1^ whereas for the other half (milk restriction) milk allowance was reduced to 6 l d^−1^ (starting after testing on day 12 with effects first assessed on day 13).

Changing the location of the baited bottles on day 13 reduced working memory (*F*_1,20_ = 12.63, *p* < 0.001), general working memory (*F*_1,20_ = 36.41, *p* < 0.001) and reference memory scores (*F*_1,20_ = 54.42, *p* < 0.001) (figure 2*a–c*). There was no effect of the feed restriction treatment on these measures, and no evidence of treatment by day interaction, but feed restriction did increase the number of vocalizations (*F*_1,20_ = 8.18, *p* = 0.01; figure 2*d*).

During the re-learning phase, feed-restricted calves showed evidence of poorer working (*F*_1,20_ = 6.62, *p* = 0.018) and general working memory (*F*_1,20_ = 6.64, *p* = 0.018), and vocalized more than non-restricted calves (*F*_1,20_ = 4.78, *p* = 0.04). We found no effect of milk restriction on reference memory (*p* > 0.05).

## 4. Discussion

Feed restriction negatively affected cognitive function in both experiments, even when baited location did not change (Experiment 1). This result is consistent with the idea that calves were distressed by the sudden milk restriction. Although, we expected calves experiencing larger declines in milk intake to experience more severe declines in cognitive performance, our results suggest that calves with lower baseline milk intakes were most affected by the decline, perhaps because they experience cumulative hunger known to be associated with stronger motivational changes [1]. Why some calves have relatively low milk intakes when offered ad libitum access to milk is unknown, but this seems to make them more vulnerable to future milk restrictions.

Although all aspects of calves' cognition were affected in Experiment 1, they were not similarly affected. Working memory proved to be particularly sensitive as it took longer before calves returned to pre-restriction performance. Calves likely habituated to the new milk allowance (reducing its emotional impact), and increased grain intakes that usually follow milk restrictions [18] may also have reduced feelings of hunger over time. Similarly, only working and general working memory differed between feed-restricted and control calves in Experiment 2; feed-restricted calves did not struggle more to learn the new baited locations but had more difficulties focusing on the task resulting in more revisits to baited and unbaited bottles. This result is consistent with previous research showing that animals with poorer welfare exhibit lower working memory when animals have to re-learn baited locations [19]. Alternatively, reduced foraging effort may explain why some calves did not perform as well once baited location changed. We think this explanation is unlikely given that this should have also impaired performance of calves in the control group, for whom getting milk from the test did not represent a high proportion of their daily intake. By contrast, our results suggest that these calves performed better than feed-restricted ones.

Although our results do not provide direct evidence that the drop in cognitive performance was emotionally driven (i.e. that calves felt too hungry to focus), the effect on cognition is consistent with the experience of distressful hunger. In addition, milk restriction increased vocalizations, a response commonly associated with negative emotions [20]. Together with previous work (e.g. [9]), our results strengthen the evidence that milk restrictions are associated with negative affective experiences in dairy calves. Given the link between cognition and emotions [21], this type of cognitive approach shows promise in enhancing our understanding of the affective experiences of animals.

## 5. Conclusion

Milk restriction negatively affects cognitive function of dairy calves, consistent with negative feelings of hunger.

## Data Availability

Data and SAS code are provided in the electronic supplementary material [17].
