# Correction to: ‘Hunger affects cognitive performance of dairy calves’ (2023) by Lecorps *et al.*

**DOI:** 10.1098/rsbl.2023.0123

**Published:** 2023-03-29

**Authors:** Benjamin Lecorps, Raphaela E. Woodroffe, Marina A. G. von Keyserlingk, Daniel M. Weary

*Biol. Lett.* **19**, 20220475. (Published online 18 January 2023) (doi:10.1098/rsbl.2022.0475)

The primary affiliation institution metadata details for author Benjamin Lecorps has been changed from the University of British Columbia to the University of Bristol. This has now been corrected on the publisher's website.

